# Dynamic Metabolite Profiling in an Archaeon Connects Transcriptional Regulation to Metabolic Consequences

**DOI:** 10.1371/journal.pone.0135693

**Published:** 2015-08-18

**Authors:** Horia Todor, Jessica Gooding, Olga R. Ilkayeva, Amy K. Schmid

**Affiliations:** 1 Department of Biology, Duke University, Durham, North Carolina, United States of America; 2 Sarah W. Stedman Nutrition and Metabolism Center, Duke Molecular Physiology Institute, Departments of Pharmacology and Cancer Biology and Medicine, Duke University Medical Center, Durham, North Carolina, United States of America; 3 University Program in Genetics and Genomics, Duke University, Durham, North Carolina, United States of America; 4 Center for Systems Biology, Duke University, Durham, North Carolina, United States of America; University of Freiburg, GERMANY

## Abstract

Previous work demonstrated that the TrmB transcription factor is responsible for regulating the expression of many enzyme-coding genes in the hypersaline-adapted archaeon *Halobacterium salinarum* via a direct interaction with a *cis*-regulatory sequence in their promoters. This interaction is abolished in the presence of glucose. Although much is known about the effects of TrmB at the transcriptional level, it remains unclear whether and to what extent changes in mRNA levels directly affect metabolite levels. In order to address this question, here we performed a high-resolution metabolite profiling time course during a change in nutrients using a combination of targeted and untargeted methods in wild-type and *ΔtrmB* strain backgrounds. We found that TrmB-mediated transcriptional changes resulted in widespread and significant changes to metabolite levels across the metabolic network. Additionally, the pattern of growth complementation using various purines suggests that the mis-regulation of gluconeogenesis in the *ΔtrmB* mutant strain in the absence of glucose results in low phosphoribosylpyrophosphate (PRPP) levels. We confirmed these low PRPP levels using a quantitative mass spectrometric technique and found that they are associated with a metabolic block in *de novo* purine synthesis, which is partially responsible for the growth defect of the *ΔtrmB* mutant strain in the absence of glucose. In conclusion, we show how transcriptional regulation of metabolism affects metabolite levels and ultimately, phenotypes.

## Introduction

The regulation of metabolism is a key challenge faced by all organisms. In unpredictable and fluctuating environments, cells must produce the same metabolic outputs to thrive. Although many central metabolic processes are well conserved across many species [1] and have been thoroughly characterized, variations of these canonical pathways and peripheral metabolic processes have not been extensively studied. The availability of high-throughput untargeted metabolomics techniques such as LC-MS/MS has led to a better understanding of the metabolic abilities and preferences of different microbes [2]. However, a complete understanding of metabolism and its regulation is still missing in many understudied organisms such as the archaea. Although archaea represent much of the microbial diversity in many environments, relatively little is known about their metabolism and its regulation. Previous work on archaeal metabolism suggests that allosteric regulation of enzymes is limited compared to the allosteric regulation of enzymes in eukaryotes and bacteria [3], suggesting an important role for transcriptional regulation. For example, haloarchaeal glutamate dehydrogenase does not respond to ADP and GDP [4], and pyruvate kinase from *H*. *salinarum*, an enzyme functioning at a central branch point in metabolism, exhibits only limited allosteric regulation [5]. Despite the importance of transcriptional regulation in controlling enzyme levels and flux through metabolism, a global analysis of the effects of transcription on metabolism has not yet been performed. Such an analysis can provide a deeper understanding of how cells respond to nutrient perturbations and how these changes affect the physiology of the organism.

Previous work identified the TrmB family of transcriptional regulators as regulators of carbon metabolism. Biochemical assays in the hyperthermophilic archaeon *Pyrococcus furiosus* demonstrated that TrmB binds DNA in the absence of its inducing sugar. Depending on the position of the TrmB *cis*-regulatory sequence relative to the transcription start site, TrmB can both activate and repress transcription [6]. A TrmB homologue in the related hyperthermophile *Thermococcus kodakarensis*, was shown to function as a global transcriptional regulator of gluconeogenic and glycolytic pathways [7]. In the hypersaline-adapted model archaeal species, *Halobacterium salinarum*, TrmB functions as a central regulator of carbon metabolism. TrmB directly regulates over 100 genes in the absence of glucose [8] and indirectly affects the expression of 182 genes. Many of these genes encode enzymes involved in diverse metabolic pathways such as gluconeogenesis, amino acid metabolism, cobalamin (vitamin B12) synthesis and purine synthesis. Despite sharing a common regulator, dynamic gene expression profiling suggests that these pathways are differentially regulated by TrmB and other regulators [9], which raises the possibility that TrmB is a global metabolic regulator. When bound to DNA, TrmB activates the expression of some genes, including genes encoding enzymes involved in gluconeogenesis and represses the expression of other genes, such as those encoding enzymes involved in glycolysis [8]. The addition of glucose abolishes TrmB-DNA binding, likely via a conformational change caused by a direct interaction with TrmB [8,10]. The abolition of TrmB binding results in de-repression of repressed genes and de-activation of previously activated genes. A strain harboring an in-frame deletion of *trmB* (*ΔtrmB*) has been generated [8]. This strain exhibits altered gene expression, impaired growth, and other abnormal physiology in the absence of glucose. Since TrmB does not bind DNA in the presence of glucose, the *ΔtrmB* mutant strain exhibits wild-type growth rate and physiology when glucose is added to the medium [8].

Intriguingly, previous work suggests that *H*. *salinarum* cannot metabolize glucose for energy. No active transport of glucose [11] has been shown; no phosphofructokinase gene or enzyme activity [12,13] has been found; glucose cannot be used as the sole carbon source [14]; and *H*. *salinarum* cannot convert ^13^C labeled glucose to ^13^C labeled pyruvate [13]. In a previous study, we addressed this contradiction by showing that TrmB transcriptionally regulates the production of sugars used to glycosylate the S-layer cell surface protein [15]. Additionally, we demonstrated that TrmB–DNA binding is well correlated with growth rate because S-layer glycosylation is proportional to growth. These observations suggested that TrmB transcriptional regulation of peripheral pathways such as purine, cobalamin, and aromatic amino biosynthesis provides a growth rate input into their specialized regulatory subnetworks [15].

Although the gene expression changes caused by TrmB and their evolutionary rationale have been explored [8,9,15], it remains unclear whether TrmB affects metabolite levels and, if so, how these changes are connected to the morphological and growth defects observed in the *ΔtrmB* mutant strain under gluconeogenic conditions. Because TrmB regulates a large and diverse regulon, understanding how mis-regulation of these genes affects metabolism can reveal novel enzymes and hitherto unrecognized connections between metabolic pathways. For these reasons, here we conducted metabolomics profiling experiments in the *ΔtrmB* mutant strain and the *Δura3* isogenic parent strain over a time course of 10 hours, which included a glucose stimulus. By interpreting the time course metabolite data in the context of previously collected dynamic gene expression and TrmB-DNA binding measurements [9], we investigated which changes in metabolite levels are caused by shifts in gene expression, and which changes in metabolite levels drive shifts in gene expression and physiology. We found that TrmB-mediated changes in gene expression are both directly and indirectly associated with significant shifts in metabolite levels across the metabolic network. Additionally, clustering of temporal metabolite patterns in both strains revealed significant enrichment of functional categories in specific clusters, suggesting that the metabolic modularity observed at the transcriptional level [9] continues at the metabolite level. In addition to a general understanding of metabolite changes, our data also provided insights into the functioning and regulation of certain pathways. Specifically, we found that the down-regulation of genes encoding gluconeogenic enzymes in the *ΔtrmB* mutant strain in the absence of glucose leads to a defect in phosphoribosylpyrophosphate (PRPP) production, which causes a metabolic block in purine biosynthesis. We present evidence suggesting that this metabolic block is responsible for the up-regulation of genes encoding enzymes involved in purine synthesis in the *ΔtrmB* mutant strain in the absence of glucose. This block in purine synthesis is partially responsible for the growth defect of the *ΔtrmB* mutant strain in the absence of glucose. Taken together, these results further both a general understanding of the effects of transcriptional regulation on metabolite level and a deeper understanding of the regulation of specific pathways.

## Results and Discussion

### TrmB-driven transcriptional regulation directly and specifically affects the levels of certain metabolites

**Fig 1:**
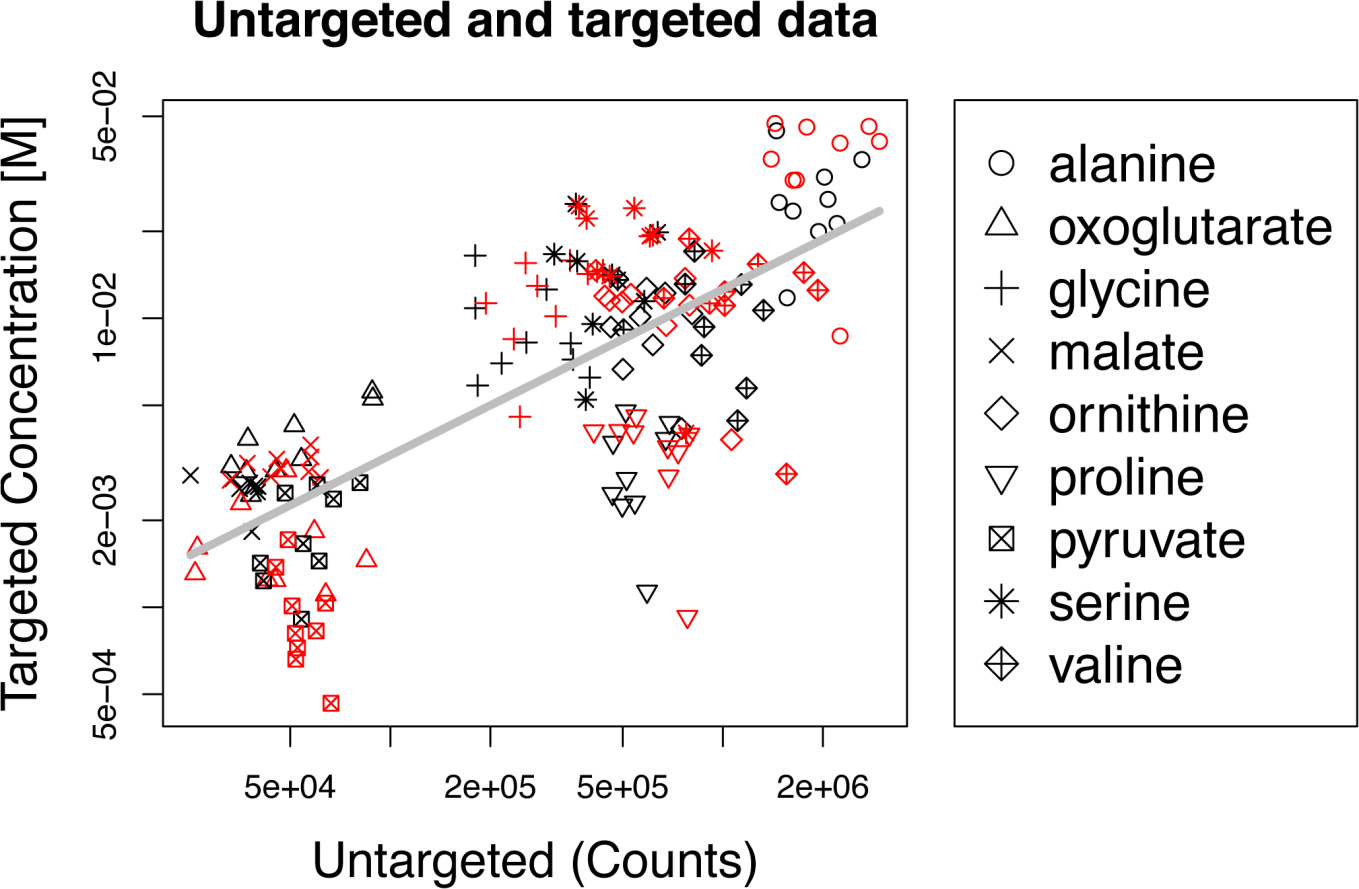
Correlation of untargeted and targeted metabolite measurements. Untargeted metabolite levels are well correlated with targeted measurements of amino acids and organic acids. Each data point on the graph corresponds to a timepoint, metabolite, and strain (n = 162; 2 strains, 9 metabolites, 9 timepoints). Each metabolite is shown using a different symbol (see legend). Detections in the Δ*ura3* strain are shown in black and detections in the Δ*trmB* strain are shown in red. Log-log linear regression of targeted and untargeted concentrations is shown in grey.

Many metabolic processes are regulated both transcriptionally and post-transcriptionally in *H*. *salinarum* and other archaea [3,9,16]. Therefore, in order to determine whether and how TrmB-mediated transcriptional regulation of enzyme-coding genes affects metabolite levels, we performed targeted and untargeted metabolomics analysis using gas chromatography (GC) and liquid chromatography (LC) coupled to mass spectrometry (MS) over a high-resolution time course in a strain harboring an in-frame deletion of *trmB* (Δ*trmB*) and its isogenic parent strain (Δ*ura3*). For collection of both targeted and untargeted data types, the *ΔtrmB* mutant and *Δura3* isogenic parent strains were grown on amino acids as the source of carbon and energy to mid-logarithmic phase (OD600 ~ 0.2–0.5) in defined medium, then sampled two or three times before and seven times after the addition of glucose (Materials and Methods). Because our untargeted protocol has not been previously validated in halophilic archaea, and because the extraction solvent chosen for an assay can bias metabolite recovery [17], we compared the untargeted metabolomics data to targeted metabolomics data for organic acids and amino acids (Materials and Methods, [18,19]). Metabolite levels obtained with the two methods were strongly and significantly correlated (Fig 1, *R* ^*2*^ > 0.55, *p* < 10^−16^), suggesting good reproducibility between different cultures, extraction solvents, and detection methods. Based on the regression fit of the untargeted GC-MS to the targeted data (Fig 1), previously quantified gamma-glutamyl cysteine levels [20], and previously quantified NAD levels [21], metabolites with intracellular concentrations in or below the single millimolar range were detected by all three of the untargeted assays (S1 Table). Taken together, these data suggest that metabolomics profiling in *H*. *salinarum* is a reproducible and sensitive method for assessing intracellular metabolite concentrations (Fig 1), and validated the untargeted data to be used in subsequent analyses.

**Fig 2:**
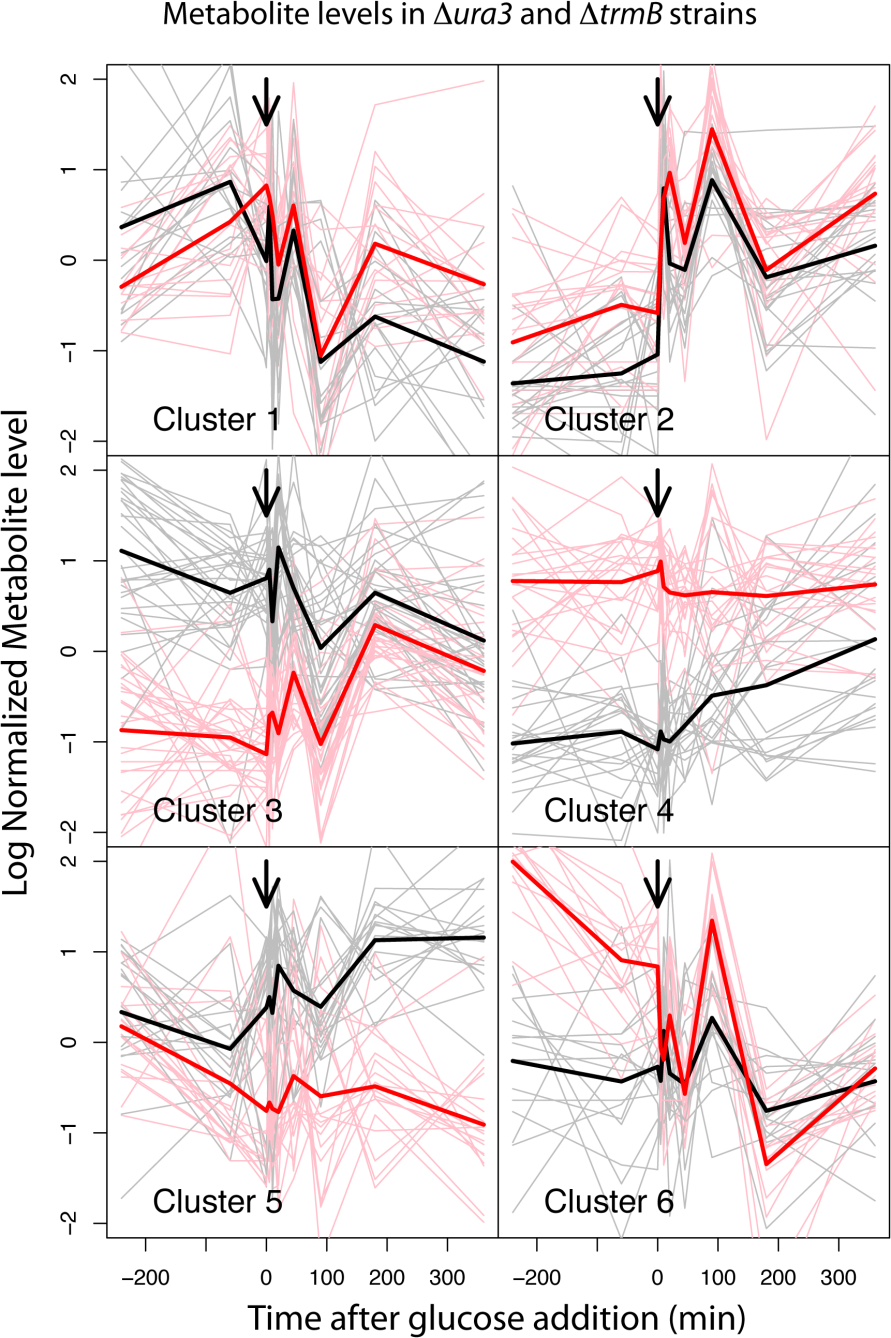
Clustering of metabolite patterns. Figure depicts six clusters of metabolite patterns. Logged, mean-scaled and normalized combined Δ*ura3* (black lines) and Δ*trmB* (red lines) data are shown. Each cluster graph depicts metabolite data for individual metabolites across 5 biological replicate cultures (thinner lines) and the mean expression profile for the cluster (thicker lines). Arrow depicts when glucose was added to a final concentration of 5%.

A total of 125 metabolites were detected in the untargeted samples. Of these, 91 were positively ascribed to known compounds and classified into 8 pathways (Table 1, S1 Table). The temporal profiles of all 125 metabolites were grouped into 6 clusters using hierarchical clustering in order to determine their response to glucose perturbation and the dependence of this response on TrmB (Fig 2, S1 Fig). We found that metabolites in specific pathways were significantly enriched in certain clusters (Table 2, S2 Table). Additionally, while some clusters showed similar patterns in the *ΔtrmB* knockout mutant and the *Δura3* isogenic parent strain, many showed distinct patterns between strains (Fig 2, S1 Fig, S2 Fig). Specifically, cluster 3, which is significantly enriched for nucleotides such as adenosine and guanosine (*p* < 0.005) and cluster 4, which is significantly enriched in peptides (*p* < 0.01) exhibited the most significant differences between the *ΔtrmB* knockout mutant and the *Δura3* isogenic parent strain in the absence of glucose but not in its presence (S2 Fig). Both cluster 3 and cluster 4 include unidentified metabolites (S2 Table), suggesting that TrmB may be involved in the regulation of unidentified metabolic pathways. In contrast to these clusters, cluster 5 metabolites exhibited a similar level between the Δ*trmB* mutant strain and the Δ*ura3* isogenic parent strain in the absence of glucose, but diverged after the addition of glucose (Fig 2, S1 Fig). This pattern may be due to different amino acids remaining in the media due to the mis-regulated metabolism of the Δ*trmB* mutant strain. Consistent with this hypothesis, many of the metabolites in cluster 5 are branched chain amino acids and their derivatives, which are known to be metabolized during midlogarithmic phase [22]. The previously observed difference in sugar levels [15] between the Δ*trmB* mutant strain and the Δ*ura3* isogenic parent strain in the absence of glucose was not recapitulated in the untargeted dynamic data (cluster 2). This is because the filter-based sample collection method used to harvest the samples for untargeted dynamic analysis resulted in ~3 fold more dilute samples than the centrifugation-based method used to collect the samples for the previous study [15], resulting in a lack of carbohydrate detections in this study prior to the addition of glucose. Specifically, prior to glucose addition only 145 out of a possible 390 peaks (13 compounds, 2 strains, 5 replicates, 3 timepoints) were detected for compounds annotated as carbohydrates (S1 Table). Nevertheless, the overall number and quantity of metabolites detected was similar across the two strains (S1 Table), ruling out the possibility that the observed differences in metabolite levels were simply due to systemic deficiencies in the Δ*trmB* mutant. Together, these data suggest that TrmB, a transcription factor regulating metabolic enzyme-coding genes [8,9], is required to maintain wild-type metabolite levels in specific pathways, including purines, peptides, and unknown pathways during nutrient perturbations.

**Table 1:** Metabolites detected using untargeted methods.

Pathway	Number
Unidentified	34
Amino acid	34
Lipids	16
Carbohydrate	13
Cofactors, Prosthetic Groups, Electron Carriers	7
Xenobiotics	7
Nucleotide	6
Peptide	6
Secondary metabolism	2

**Table 2:** Significance of enrichment of functional categories in clusters of metabolite patterns.

Cluster	Pathway	Corrected *p*-value
**Cluster 1**	N/A	6.65E-06
**Cluster 2**	Carbohydrate	6.08E-09
**Cluster 3**	Xenobiotics	0.000901
	Nucleotide	0.003562
	Secondary metabolism	0
**Cluster 4**	Peptide	0.008786
**Cluster 5**	Lipids	0.00306
	Peptide	0.004867
**Cluster 6**	Amino acid	1.90E-06

### Temporal dynamics of growth recovery

**Fig 3:**
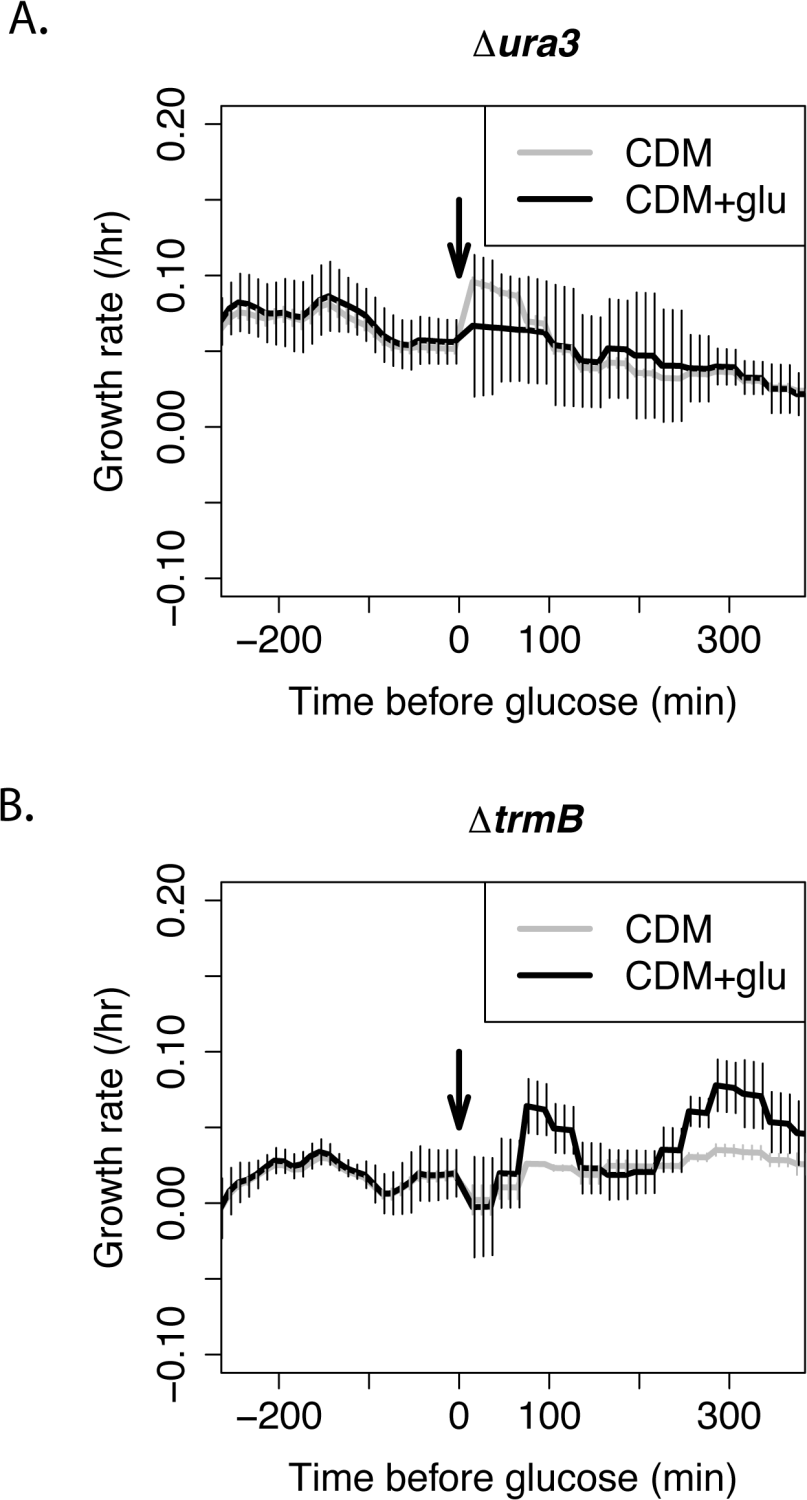
Growth rate during glucose addition. Instantaneous growth rate of the Δ*ura3* parent strain (A) and Δ*trmB* mutant strain (B) during the glucose addition (black lines) and control (grey lines) time course. Cells were grown in Complete Defined Medium (CDM). Error bars represent the standard error from the mean of 3 biological replicate cultures.

In order to understand how changes in metabolite levels affect the physiology of *H*. *salinarum* before and after the addition of glucose, we sampled the optical density of the cultures of the *ΔtrmB* mutant strain and the *Δura3* isogenic parent strain every 10 minutes before, during, and after the addition of glucose during mid-log growth in CDM (Materials and Methods). We calculated the instantaneous growth rate during each of these 10 minute periods in order to determine how growth rate changes after the addition of glucose. The addition of glucose to the *Δura3* isogenic parent did not appear to cause a significant change in growth rate (Fig 3A, S3 Fig). In contrast, the growth rate of the *ΔtrmB* mutant strain is low prior to the addition of glucose, but recovers to a level similar to that of the *Δura3* isogenic parent strain approximately 200 minutes after glucose addition (Fig 3B, S3 Fig).

In order to determine which metabolites may be linked to changes in growth rate, we calculated the correlation between the level of each metabolite at each time point in each strain and the instantaneous growth rate at those time points in those strains. Seven of the 125 metabolites were significantly anticorrelated with growth rate, including three gamma-glutamyl amino acids as well as unidentified metabolites (Table 3). Based on the presence of many gamma-glutamyl amino acids as well as 5-oxoproline in our data set, it appears that *H*. *salinarum* possesses a functional gamma-glutamyl cycle [23]. Although the function of this gamma-glutamyl cycle in the haloarchaea remains to be uncovered, gamma-glutamylcysteine, a key intermediate, was found to be the major low molecular weight thiol in *H*. *salinarum* [20], functioning like glutathione to buffer redox stress. Additionally, gamma-glutamylcysteine was shown to be required for tolerance of certain redox stresses in the related halophile *Haloferax volcanii* [24]. These observations suggest that the anticorrelation between gamma-glutamyl amino acid levels and growth rate may be due to increased activity of the gamma-glutamyl cycle related to the redox imbalance experienced by the *ΔtrmB* mutant strain in the absence of glucose [8], although further experiments are required to test this possibility. Five of the 125 metabolites detected were significantly correlated with instantaneous growth rate, with the metabolite profiles matching the growth profiles in both strain backgrounds (Table 3). These included purine compounds (e.g. adenosine, guanosine, NAD^+^), suggesting that a defect in purine metabolism may contribute to the growth defect of the *ΔtrmB* knockout mutant strain.

**Table 3:** Metabolites significantly correlated with growth rate in the *ΔtrmB* mutant strain and the *Δura3* isogenic parent strain.

	Correlation	R^2^	Corrected *p*-Value
X - 16071	Negative	0.5128	4.800E-02
X - 16682	Negative	0.5763	1.300E-02
N-acetylthreonine	Negative	0.5927	9.000E-03
tryptophanol	Negative	0.6294	3.800E-03
gamma-glutamylvaline	Negative	0.6441	2.600E-03
gamma-glutamylalanine	Negative	0.668	1.400E-03
gamma-glutamyl-2-aminobutyrate	Negative	0.7107	4.000E-04
X - 20525	Positive	0.5379	2.920E-02
NAD+	Positive	0.5915	9.200E-03
adenosine	Positive	0.6444	2.600E-03
guanosine	Positive	0.6667	1.400E-03
N-carbamoylaspartate	Positive	0.6687	1.300E-03

### Purine synthesis is negatively regulated by purine levels

**Fig 4:**
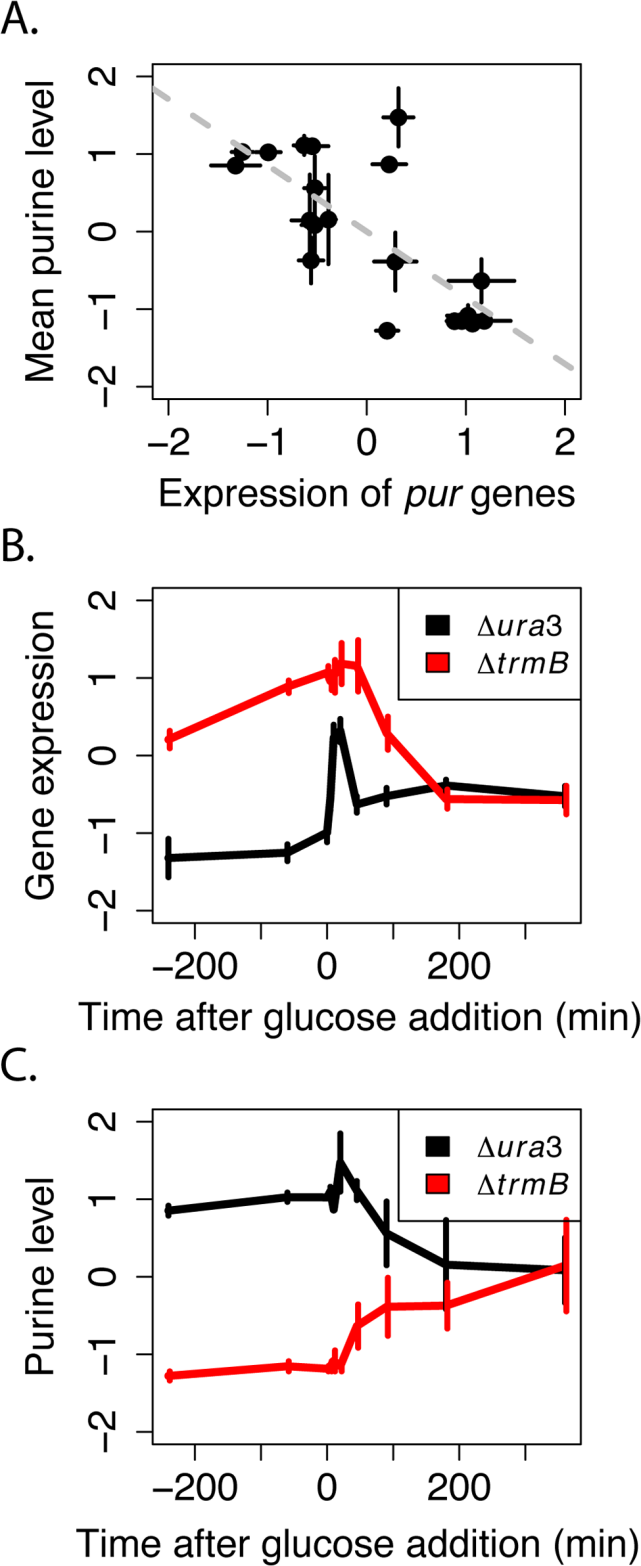
Levels of purine metabolites and purine synthesis genes. (A) Expression of genes encoding enzymes involved in *de novo* purine synthesis is negatively correlated to intracellular purine levels. Each point represents the average of the log-transformed, mean-centered and normalized gene expression in purine synthesis genes (X-axis, Materials and Methods, [9]) plotted against the average of the log-transformed, mean-centered and normalized level of adenosine and guanosine (Y-axis, Materials and Methods) in the Δ*ura3* parent strain and Δ*trmB* mutant strain at each timepoint (n = 20; ten timepoints, two strains). Error bars represent standard error from the mean of the 11 genes or the 2 metabolites. Dotted trendline represents the linear regression between purine level and the expression of genes encoding enzymes involved in purine synthesis. (B) Temporal profile of the expression of genes encoding enzymes involved in purine synthesis (Materials and Methods, [9]) over the glucose stimulus time course in the Δ*ura3* (black lines) and Δ*trmB* (red lines) strains. (C) Temporal profile of purine levels over the metabolomics time course in the Δ*ura3* (black lines) and Δ*trmB* (red lines) strains (Materials and Methods).

Since TrmB is a transcriptional regulator that has been shown to bind near the promoter of some of genes encoding enzymes involved in *de novo* purine synthesis [8], we reasoned that the defect in purine levels we observed in the *ΔtrmB* mutant in the absence of glucose (Fig 2, Cluster 3, S1 Fig) may be due to mis-regulation of genes encoding purine synthesis enzymes due to the lack of TrmB. In order to address this question, the expression levels of genes encoding enzymes involved in purine biosynthesis (data from [9]) were compared to the purine metabolite levels (Fig 4, Materials and Methods) in both the *ΔtrmB* mutant strain and its isogenic *Δura3* parent strain. The previously published expression data were collected over the same time course and growth conditions as the metabolite levels measured here, so these data could be compared at each time point. A strong negative correlation (*R* ^*2*^ *>* 0.55, *p <* 10^−3^, Fig 4A) was detected between gene expression and purine level across both strains and nutrient conditions. This suggests that, although previous high throughput TF-DNA binding experiments detected weak TrmB binding at purine biosynthesis genes [8], TrmB plays an indirect role in regulating the expression of genes encoding enzymes involved in purine synthesis, consistent with insights gleaned from dynamic gene expression profiles [9]. The negative correlation observed is consistent with the hypothesis that the transcription of purine synthesis genes is negatively regulated by the availability of purines through a different transcription factor or another mechanism, similar to what has been observed in *Escherichia coli* and *Saccharomyces cerevisiae* [25,26] and consistent with inferences from our dynamic transcriptomic data [9]. Interpreted in this light, the elevated expression of genes coding for enzymes involved in purine biosynthesis and the lack of purines in the *ΔtrmB* strain in the absence of glucose suggest a metabolic block in *de novo* purine biosynthesis (Fig 4B and 4C), perhaps caused by the role of TrmB in regulating gluconeogenesis to produce precursors to purine biosynthesis [27].

### A defect in purine synthesis is partially responsible for the *ΔtrmB* growth defect under gluconeogenic conditions

**Fig 5:**
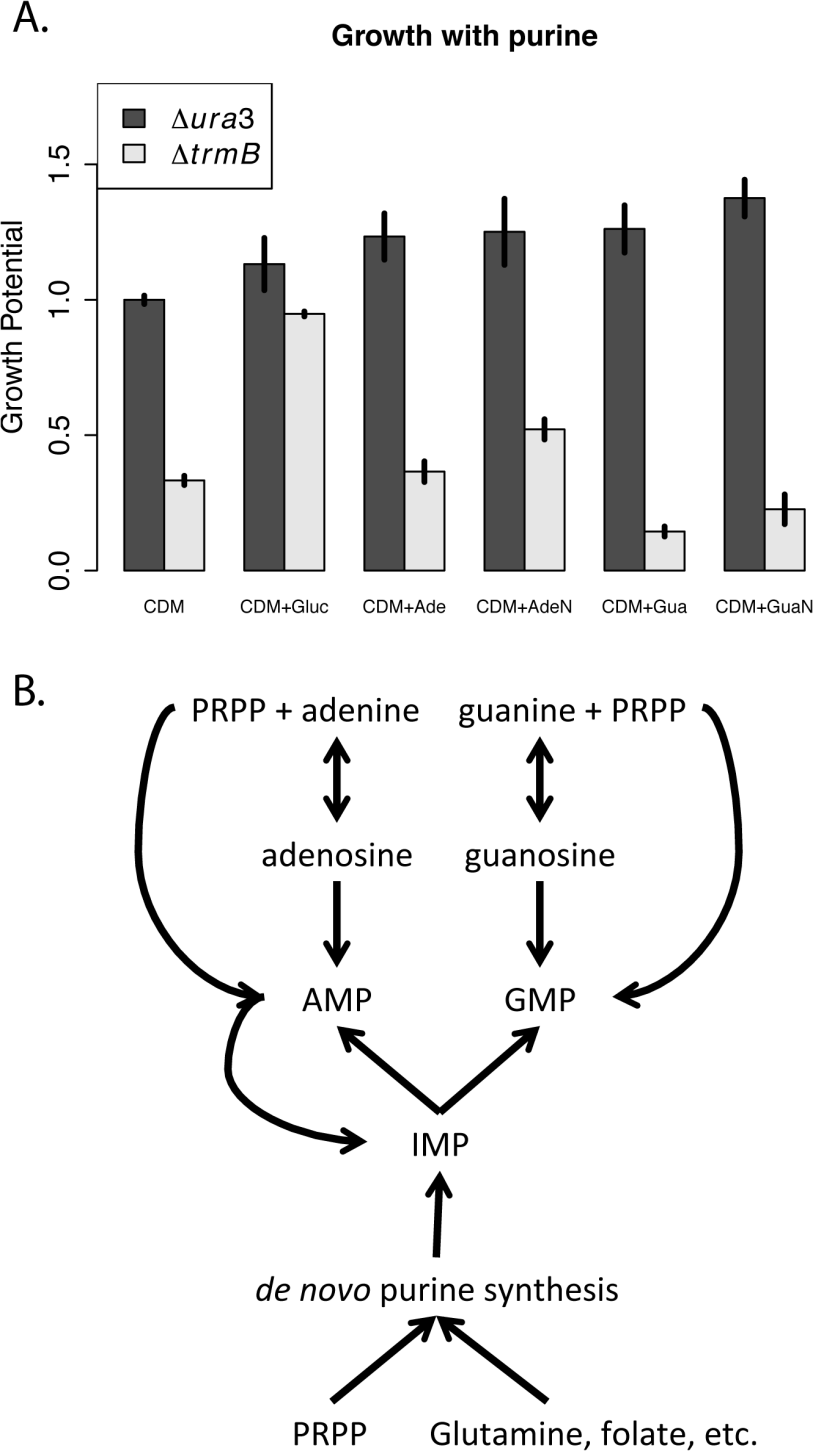
Growth complementation with purine compounds. (A) The average growth potential (Materials and Methods) of the Δ*ura3* parent strain (dark bars) and Δ*trmB* mutant strain (light bars) in complete defined medium (CDM), CDM supplemented with 5% glucose (CDM+ Gluc), and CDM supplemented with 100 μM of adenine (CDM+Ade), adenosine (CDM+AdeN), guanine (CDM+Gua), and guanosine (CDM+GuaN). Error bars represent the standard error from the mean of 7 biological replicate cultures. (B) Simplified diagram of purine salvage in *H*. *salinarum* adapted from [21].

In order to test the hypothesis that the growth defect of the *ΔtrmB* mutant strain in the absence of glucose, Fig 3) may be caused by inhibited purine synthesis due to a metabolic block, we assayed the growth of the *ΔtrmB* mutant and its isogenic parent strain *Δura3* in response to purine addition (Materials and Methods). Previous work has shown that purines are readily taken up by *H*. *salinarum* and integrated into biomass and nucleic acids [21]. Adenosine significantly, but incompletely, complemented the growth defect of the *ΔtrmB* strain in the absence of glucose (*p* < 0.01, Fig 5A, Table 4). Once taken up by the cell, adenosine is converted to inosine monophosphate (IMP). IMP can be converted to either GMP or AMP. These nucleotides can then be assimilated into RNA, phosphorylated to tri-nucleotides and otherwise function in the cell (Fig 5B, 21). Although adenosine partially complemented the growth defect of the *ΔtrmB* strain in the absence of glucose, adenine did not (Fig 5A, Table 4). Since the enzymatic activity of adenine phosphoribosyltransferase, which uses PRPP to phosphoribosylate adenine into AMP (Fig 5B), has been detected in *H*. *salinarum* [21], and since the gene encoding adenine phosphoribosyltransferase (*VNG0559G*) is expressed at the same level in both strains regardless of glucose level (S4 Fig, [8]), these data suggest that there is insufficient PRPP present to phosphoribosylate the adenine into AMP to complement the growth defect. Guanosine was also unable to complement the growth defect of the *ΔtrmB* strain in the absence of glucose (Fig 5A, Table 4), likely because *H*. *salinarum* lacks GMP reductase activity, which is required to convert GMP from guanosine to AMP [21]. Interestingly, supplementation with guanine causes no change in the growth of the *Δura3* isogenic parent strain, but appears to adversely affect the growth of the *ΔtrmB* knockout (*p <* 4x10^-5^, Fig 5A, Table 4). This observation is consistent with guanine addition further decreasing PRPP levels (and hence AMP levels) via increased competition for PRPP by guanine phosphoribosyltransferase. Since GMP cannot be converted to AMP, this would result in a decrease in effective purine concentration if PRPP concentration is limiting. Taken together, these data suggest that PRPP levels are depressed in the *ΔtrmB* mutant in the absence of glucose. Since PRPP is required in the absence of purine supplementation for the initial step of *de novo* purine biosynthesis, a defect in PRPP contributes to the defect in purine levels in the *ΔtrmB* mutant in the absence of glucose, partially contributing to the Δ*trmB* growth defect.

**Table 4:** Bonferroni corrected *p*-values of the T-test of difference in growth compared to complete defined medium (CDM) without additives.

Condition	*Δura3*	*ΔtrmB*
**CDM+Gluc**	1	4.95E-10
**CDM+Ade**	0.170055	1
**CDM+AdeN**	0.430323	0.007913
**CDM+Gua**	0.119496	3.96E-05
**CDM+GuaN**	0.006139	0.526128

### PRPP is found at a low level in *ΔtrmB* mutant strain in the absence of glucose

**Fig 6:**
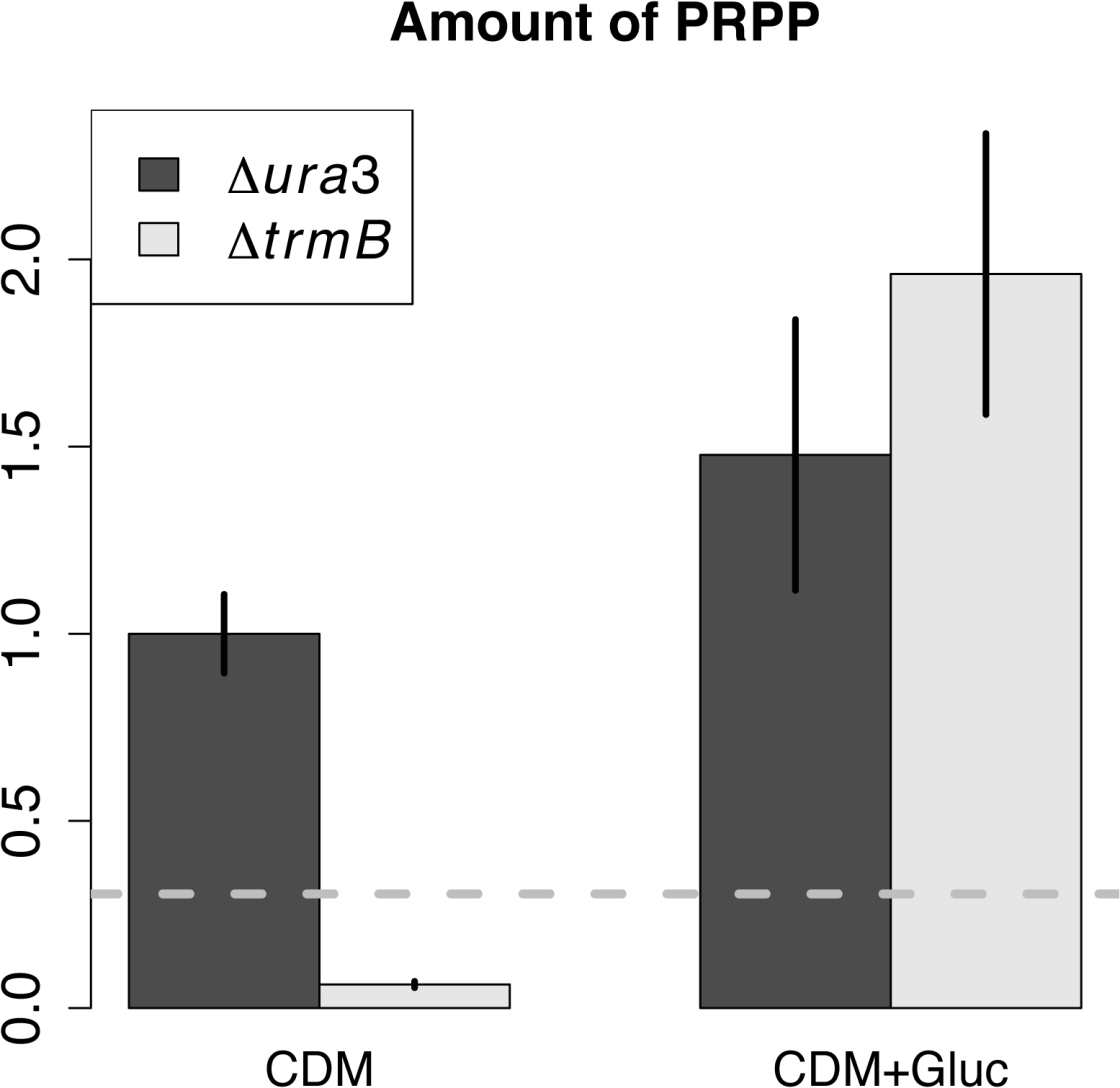
PRPP levels in the Δ*ura3* and Δ*trmB* strains with and without glucose. PRPP levels detected in the Δ*ura3* parent strain (dark bars) and the Δ*trmB* mutant strain (light bars). Error bars represent the standard error from the mean of 8 biological replicate cultures. Detection limit calculated from a standard curve is shown as a dashed grey line.

PRPP and most other polar compounds were not detected in our untargeted metabolomics studies due to diversion of the first minute of the LC column to avoid buildup of salt inside the instrument. Therefore, in order to determine if PRPP limitation is associated with the growth defect of the *ΔtrmB* mutant growing in the absence of glucose, we assayed PRPP levels using a targeted LC-MS method [28] in the *ΔtrmB* mutant strain and its *Δura3* isogenic parent strain in the presence and absence of glucose. We detected PRPP in the *ΔtrmB* mutant strain with glucose and *Δura3* isogenic parent strain with or without glucose but not in the *ΔtrmB* mutant strain in the absence of glucose, suggesting that PRPP levels were significantly lower in the *ΔtrmB* mutant strain grown without glucose (Fig 6). Taken together with the gene expression and growth complementation data (Figs 4 and 5), this observation strongly suggests that a deficiency in PRPP, the precursor of *de novo* purine synthesis, contributes partially to the growth defect of the *ΔtrmB* mutant strain in the absence of glucose.

**Fig 7:**
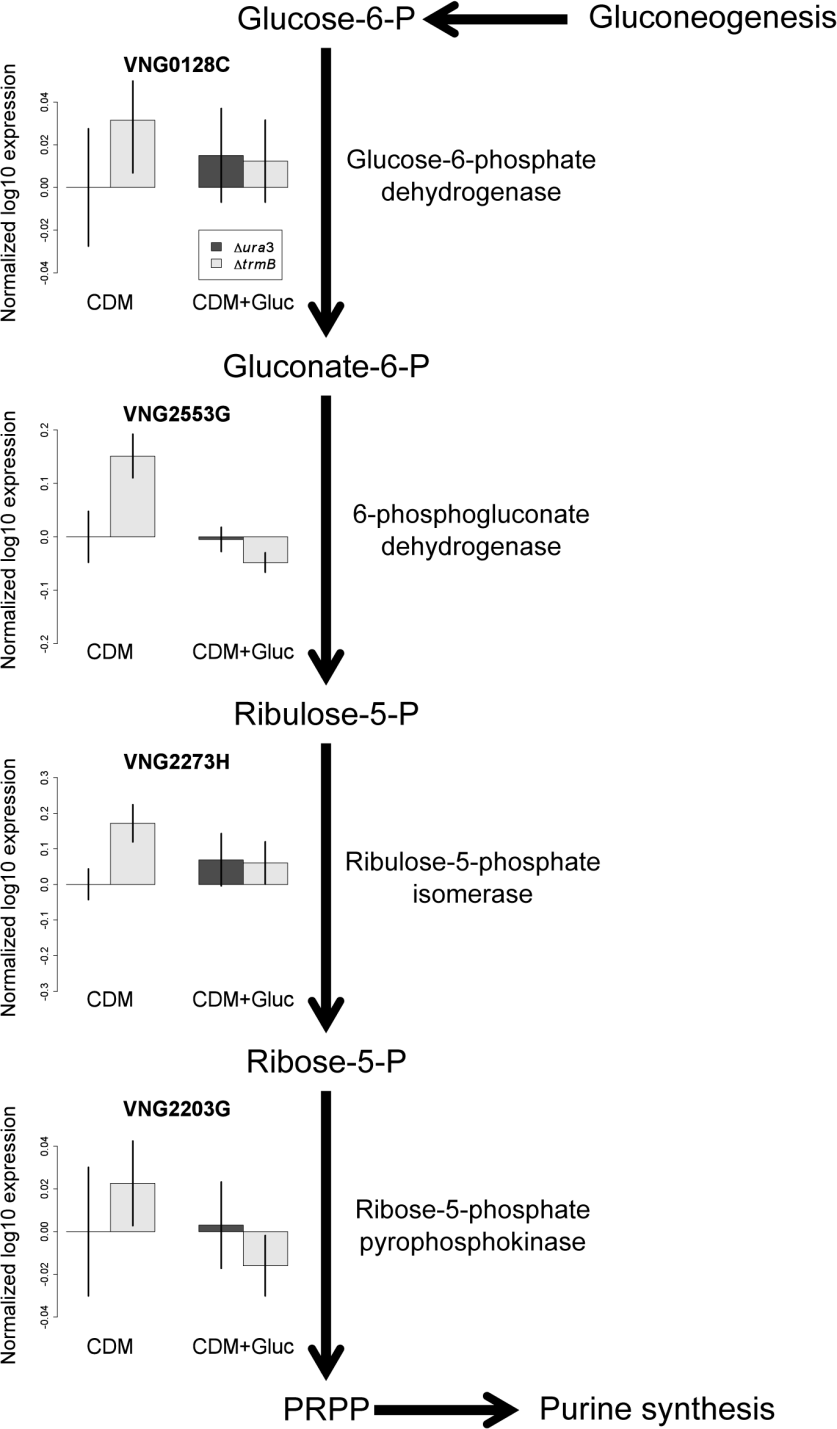
Expression of OPPP enzyme-coding genes in the Δ*ura3* and Δ*trmB* strains with and without glucose. The expression of genes encoding enzymes involved in the OPPP is not deficient in the Δ*trmB* mutant in the absence of glucose. Bar plots show log10 gene expression normalized to the Δ*ura3* parent strain in the absence of glucose for genes encoding each step in the OPPP (data from [8]) in the Δ*ura3* parent strain (dark bars) and Δ*trmB* mutant strain (light bars) in the absence and presence of glucose. Error bars represent standard error from the mean of at least 5 microarrays.

Recent work suggests that halophiles generate PRPP via a modified oxidative pentose phosphate pathway (OPPP) proceeding from glucose-6-phosphate, to 6-phosphogluconate, ribulose-5-phosphate, ribose-5-phosphate and ultimately to PRPP [29]. In order to determine which of these steps is inhibited in the *ΔtrmB* mutant strain in the absence of glucose, we compared the expression level of the genes encoding the enzymes involved in each step of the pathway in both the *Δura3* and *ΔtrmB* mutant strains in the presence and absence of glucose from previously collected transcriptomic data ([9], Fig 7). The expression of all genes encoding enzymes in the OPPP was either significantly higher or the same in the *ΔtrmB* mutant strain in the absence of glucose compared to the Δ*ura3* parent strain and the Δ*trmB* strain in the presence of glucose (Fig 7). These observations suggest that the lack of PRPP in the *ΔtrmB* mutant strain in the absence of glucose is due to insufficient levels of glucose-6-phosphate rather than transcriptional downregulation of the OPPP. This hypothesis is consistent with the role of TrmB as the sole, direct activator of genes encoding enzymes involved in gluconeogenesis [8,9] required to maintain sufficient glucose levels as well as with previously described deficiency in sugar levels in the *ΔtrmB* mutant strain in the absence of glucose [15].

## Conclusions

Understanding the regulation of metabolism in archaea remains a central challenge to successfully tapping the vast reservoir of pathways and compounds in these organisms for industrial applications. Previous studies have identified TrmB as a central transcriptional regulator of metabolism in *H*. *salinarum*. In order to understand how transcriptional regulation of enzyme coding genes affects metabolism, we performed targeted and untargeted measurements of the metabolome of *H*. *salinarum* during a change in nutrient conditions. We found that the transcriptional regulation causes substantial changes in intracellular metabolite levels, which cause changes in physiology. Specifically, we identified a metabolic block in *de novo* purine synthesis in the *ΔtrmB* mutant strain in the absence of glucose, which is partially responsible for the observed growth rate defect. Combining dynamic metabolite and gene expression data, we showed that this block was due to a defect in PRPP caused by mis-regulation of genes encoding enzymes involved in gluconeogenesis and therefore a lack of OPPP substrates. Taken together, these data suggest that transcriptional regulation of enzyme coding genes is an important mechanism for regulating metabolism.

## Materials and Methods

### Strains

*Halobacterium salinarum* NRC-1 (ATCC strain 700922) was used as the wild-type strain background. Experiments were performed in two previously constructed strains, one containing an in-frame deletion of *VNG1451C* (Δ*ura3ΔtrmB*, [8]) and its isogenic parent strain, Δ*ura*3 [30].

### Growth conditions and growth rate calculation

Cells were grown routinely in Complete Defined Medium (CDM) containing 19 amino acids [9] as indicated in the figures. The medium was supplemented with 50 μg ml^-1^ uracil to complement the *ura3* deletion. Cultures were routinely grown at 42°C while shaking at 225 r.p.m. under low ambient light.

In order to assess instantaneous growth rate during glucose addition, 200 μl cultures of the *ΔtrmB* mutant strain and its isogenic parent strain Δ*ura*3 were grown at 42°C under continuous shaking (~225 r.p.m.) in a Bioscreen C automated growth curve analysis system (Growth Curves USA, Piscataway, NJ) to mid-logarithmic phase. Glucose, sucrose control, or nothing was added to the wells once mid-logarithmic phase was reached. Optical density at 600 nm was measured every 10 minutes for each sample during the duration of the experiment. Instantaneous growth rate was calculated from the log transformed and LOWESS-smoothed data using the bsd Analysis Function previously described [15]. In order to account for osmotic effects, instantaneous growth rate in the cultures to which glucose was added was calculated by adding the difference of the glucose and sucrose instantaneous growth rates at each time point to the growth rate of the untreated control.

To measure the growth of the *ΔtrmB* mutant strain and its isogenic parent strain Δ*ura*3 in CDM supplemented with 100 μM concentration of the purines indicated in Fig 5, 200 μl cultures were grown in a Bioscreen C as described above except that optical density at 600 nm was measured every 30 minutes. Area under the log-transformed growth curve (growth potential) was used as the growth metric to convey information about both growth rate and carrying capacity of the culture. These calculations are packaged into the bsd Analysis Function previously described [15].

### Collection and quantification of metabolomics time course samples

Cells were grown to mid-logarithmic phase (OD ~ 0.3) in CDM as described above. For metabolomics time courses, 10 ml aliquots were removed from the continuously shaking cultures of both the *Δura3* and *ΔtrmB* strains at time points as indicated in Table 5. Cells were immediately filtered using a Millipore SteriFlip 0.22 μm filter and washed with 10 ml basal salts media. Since *H*. *salinarum* lyses readily in methanol and other solvents because of its requirement for high salinity, cells were lysed on the membrane filter using 1 ml of ice-cold 80% methanol with extraction standards, ice-cold 100% methanol with extraction standards, or 1 ml 50% acetonitrile with 0.3% Formic acid as indicated (Table 5). Samples were immediately snap frozen at -80°C. Untargeted samples were analyzed at Metabolon Corp. (Durham, NC), and targeted samples were analyzed at the Stedman Center for Nutrition and Metabolism, Duke Molecular Physiology Institute (Durham, NC) using previously validated methods (Table 5; [18, 19, 28, 31]). For both the untargeted samples measured at Metabolon and the samples used for the purine panel, the first minute of the LC column was diverted to avoid salt buildup in the instrument.

**Table 5:** Summary of metabolite extraction and quantitation methods.

Method	Time Points Analyzed	Replicates	Method Details	Extraction Solvent	Analysis Reference
Targeted OA Panel	-60, 0, 5, 10, 20, 45, 90, 180, 360	Δ*ura3–*6 replicates, Δ*trmB–* 2 replicates	Organic acids were quantified using methods described previously [19] employing Trace Ultra GC coupled to ISQ MS operating under Xcalibur 2.2 (Thermo Fisher Scientific, Austin, TX).	50% Acetonitrile + 0.3% Formic Acid	[19]
Targeted AA Panel	-60, 0, 5, 10, 20, 45, 90, 180, 360	Δ*ura3–*6 replicates, Δ*trmB–* 2 replicates	Amino acids were quantified by flow injection tandem mass spectrometry using sample preparation methods described [18]. Data were acquired using a Waters Acquity UPLC system equipped with a TQ (triple quadrupole) detector and a data system controlled by MassLynx 4.1 operating system (Waters, Milford, MA).	50% Acetonitrile + 0.3% Formic Acid	[18]
Untargeted LC/MS pos	-240, -60, 0, 5, 10, 20, 45, 90, 180, 360	5 replicates	Measurements were performed by UPLC-MS/MS (positive mode) using a Waters Acquity UPLC with an acidic mobile phase (solvent A: 0.1% formic acid in H2O, solvent B: 0.1% formic acid in methanol) on a 2.1 × 100 mm Waters BEH C18 1.7 μm particle column and a Thermo-Finnigan linear trap quadrupole mass spectrometer as described previously [31].	80% Methanol	[31]
Untargeted LC/MS neg	-240, -60, 0, 5, 10, 20, 45, 90, 180, 360	5 replicates	Measurements were performed by UPLC-MS/MS (positive mode) using a Waters Acquity UPLC with a basic mobile phase (solvent A: 6.5 mM ammonium bicarbonate pH 8.0, solvent B: 6.5 mM ammonium bicarbonate in 98% methanol) on a 2.1 × 100 mm Waters BEH C18 1.7 μm particle column and a Thermo-Finnigan linear trap quadrupole mass spectrometer as described previously [31].	80% Methanol	[31]
Untargeted GC/MS	-240, -60, 0, 5, 10, 20, 45, 90, 180, 360	5 replicates	Measurements were performed on a Thermo-Finnigan Trace DSQ fast-scanning single-quadrupole mass spectrometer. Electron impact ionization at 70 eV was used and the column temperature was ramped between 60 and 340°C with helium as carrier gas as previously described [31].	80% Methanol	[31]
Purine Panel	+Glucose,-Glucose	8 replicates	Samples were quantified as previously described [28] using a reverse-phase ion-pairing method. PRPP was detected by the fragmentation of 389 m/z to 177 m/z at a fragmentor voltage of 105 V and collision energy of 18 V.	100% Methanol	[28]

### Normalization, outlier removal, and clustering of untargeted data

Each sample from the untargeted GC and LC-MS data was normalized by the coefficients of a linear regression between its log-transformed values and the mean of all log-transformed samples. Outlier removal was performed after normalization using Dixon’s test with a *p*-value cutoff of 0.05 for each metabolite in each strain at each time point. Remaining data were averaged for each time point, strain and metabolite. For a given time point, when a metabolite was not detected in any of the 5 replicates, the minimum value of the metabolite from the remaining data was used as the value for that time point. The log-transformed scaled and centered metabolite measurements were clustered by hierarchical clustering with 6 clusters using the complete linkage method. P-values of significant differences between Δ*ura3* parent strain and the Δ*trmB* mutant strain were calculated using a Welch’s two sided T-test at each timepoint for each cluster (S2 Fig). Enrichment within each cluster was calculated using a Bonferroni corrected hypergeometric test on the categories provided by Metabolon for each metabolite.

### Analysis of purine metabolites and purine genes

The log-transformed, mean-centered and normalized time course gene expression data [9] for genes encoding enzymes involved in purine synthesis (*prsA*, *purB*, *purC*, *purD*, *purE*, *purF*, *purH*, *purK*, *purl*, *purL2*, *purM*, *purU*, *VNG2371C*) were averaged at each time point during the glucose addition time course in the *ΔtrmB* mutant strain and *Δura3* parent strain (n = 20). These measurements were correlated using a linear model to the averaged log-transformed mean-centered and normalized data for guanosine and adenosine measured at the same time points.

## Supporting Information

S1 Fig Heatmap representation of metabolite patterns. Figure depicts six clusters of metabolite patterns. Logged, mean-scaled and normalized combined Δ*ura3* (right) and Δ*trmB* (left) data are shown. Cluster 1 is shown in black, cluster 2 is shown in red, cluster 3 is shown in blue, cluster 4 is shown in green, cluster 5 is shown in orange and cluster 6 is shown in yellow.
(PDF)

S2 Fig *p*-values of difference between the Δ*ura3* and Δ*trmB* strains for each of the six clusters. Figure depicts the negative log10 *p*-value (higher value is more significant) of the T-test between metabolite levels in the Δ*ura3* and Δ*trmB* strains for each of the six clusters of metabolite patterns at each time point. Arrow depicts when glucose was added to a final concentration of 5%.
(PDF)

S3 Fig Optical density over the course of glucose additon. Mean optical density of 3 biological replicate cultures of the Δ*ura3* parent strain (A) and Δ*trmB* mutant strain (B) during the glucose addition (black lines), control (dark grey lines), and sucrose control (light grey lines) time course. Cells were grown in Complete Defined Medium (CDM).
(PDF)

S4 Fig Expression of the gene encoding adenine phosphoribosyltransferase in the Δ*ura3* and Δ*trmB* strains with and without glucose. The expression of the gene encoding adenine phosphoribosyltransferase is similar in both the Δ*trmB* mutant and its Δura3 isogenic parent strain in the absence or presence of glucose. Bar plots show log10 gene expression normalized to the Δ*ura3* parent strain in the absence of glucose (data from [8]) in the Δ*ura3* parent strain (dark bars) and Δ*trmB* mutant strain (light bars) in the absence and presence of glucose. Error bars represent standard error from the mean of at least 5 microarrays.
(PDF)

S1 Table Raw and normalized untargeted metabolite data used in the study. (XLSX)

S2 Table Metabolites in each of the six clusters. (XLSX)
