# Supplementary material for: Dynamic Metabolite Profiling in an Archaeon Connects Transcriptional Regulation to Metabolic Consequences
Source: PLoS One. 2015 Aug 18;10(8):e0135693. doi: 10.1371/journal.pone.0135693 (PMC4540570; doi:10.1371/journal.pone.0135693)
Supplement: S1 Fig — Figure depicts six clusters of metabolite patterns. Logged, mean-scaled and normalized combined Δura3 (right) and ΔtrmB (left) data are shown. Cluster 1 is shown in black, cluster 2 is shown in red, cluster 3 is shown in blue, cluster 4 is shown in green, cluster 5 is shown in orange and cluster 6 is shown in yellow. (PDF) [file pone.0135693.s001.pdf]

Supplementary Figure 1

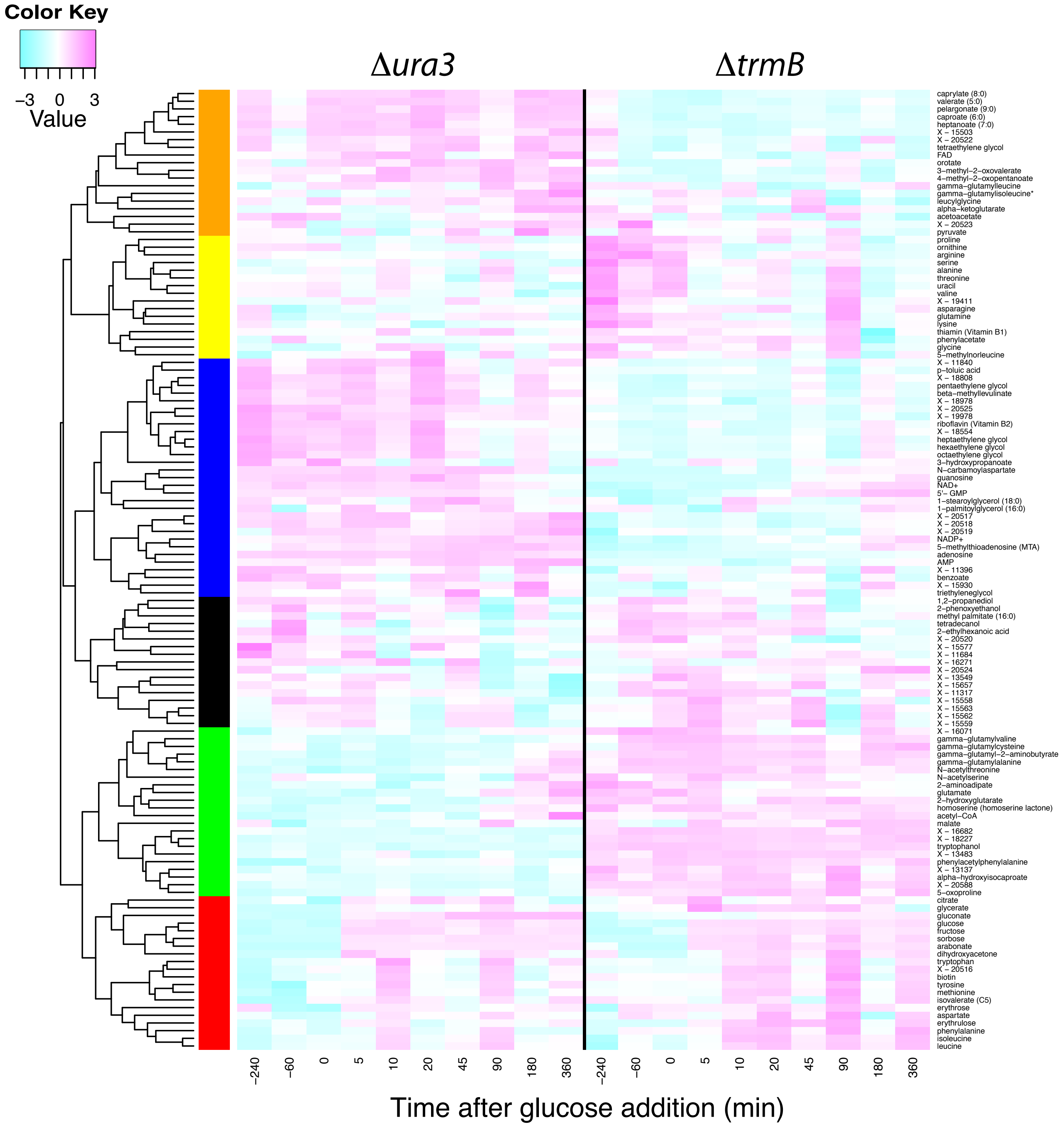

Supplementary Figure 1. Heatmap representation of metabolite patterns. Figure depicts six clusters of metabolite patterns. Logged, mean-scaled and normalized combined Δ*ura3* (right) and Δ*trmB* (left) data are shown. Cluster 1 is shown in black, cluster 2 is shown in red, cluster 3 is shown in blue, cluster 4 is shown in green, cluster 5 is shown in orange and cluster 6 is shown in yellow.
