# Supplementary material for: Dynamic Metabolite Profiling in an Archaeon Connects Transcriptional Regulation to Metabolic Consequences
Source: PLoS One. 2015 Aug 18;10(8):e0135693. doi: 10.1371/journal.pone.0135693 (PMC4540570; doi:10.1371/journal.pone.0135693)
Supplement: S2 Fig — Figure depicts the negative log10 p-value (higher value is more significant) of the T-test between metabolite levels in the Δura3 and ΔtrmB strains for each of the six clusters of metabolite patterns at each time point. Arrow depicts when glucose was added to a final concentration of 5%. (PDF) [file pone.0135693.s002.pdf]

Supplementary Figure 2

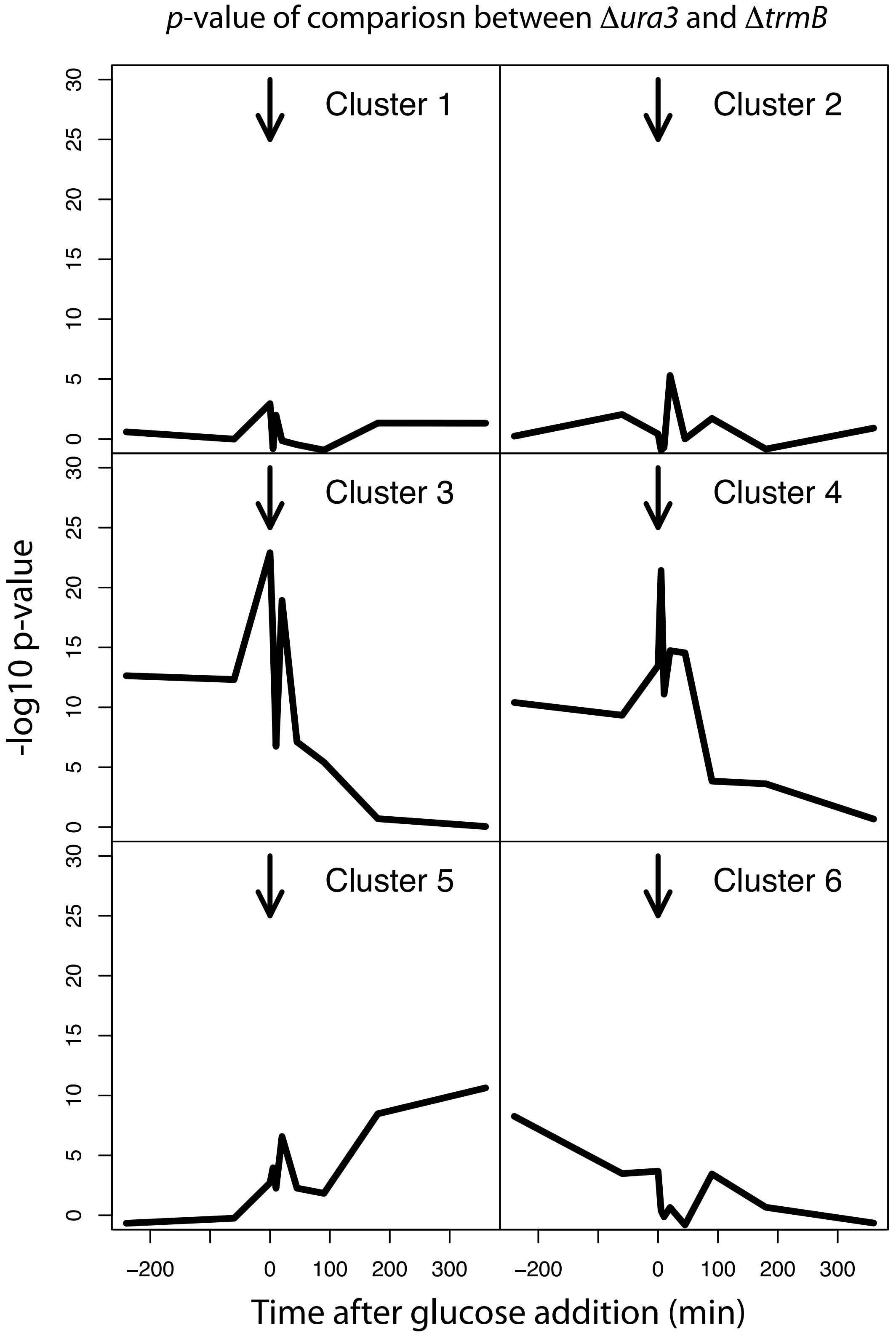

Supplementary Figure 2. Figure depicts the negative log10 p-value (higher value is more significant) of the T-test between metabolite levels in the Δ*ura3* and Δ*trmB* strains for each of the six clusters of metabolite patterns at each time point. Arrow depicts when glucose was added to a final concentration of 5%.
