# Supplementary material for: Dynamic Metabolite Profiling in an Archaeon Connects Transcriptional Regulation to Metabolic Consequences
Source: PLoS One. 2015 Aug 18;10(8):e0135693. doi: 10.1371/journal.pone.0135693 (PMC4540570; doi:10.1371/journal.pone.0135693)
Supplement: S3 Fig — Mean optical density of 3 biological replicate cultures of the Δura3 parent strain (A) and ΔtrmB mutant strain (B) during the glucose addition (black lines), control (dark grey lines), and sucrose control (light grey lines) time course. Cells were grown in Complete Defined Medium (CDM). (PDF) [file pone.0135693.s003.pdf]

Supplementary Figure 3

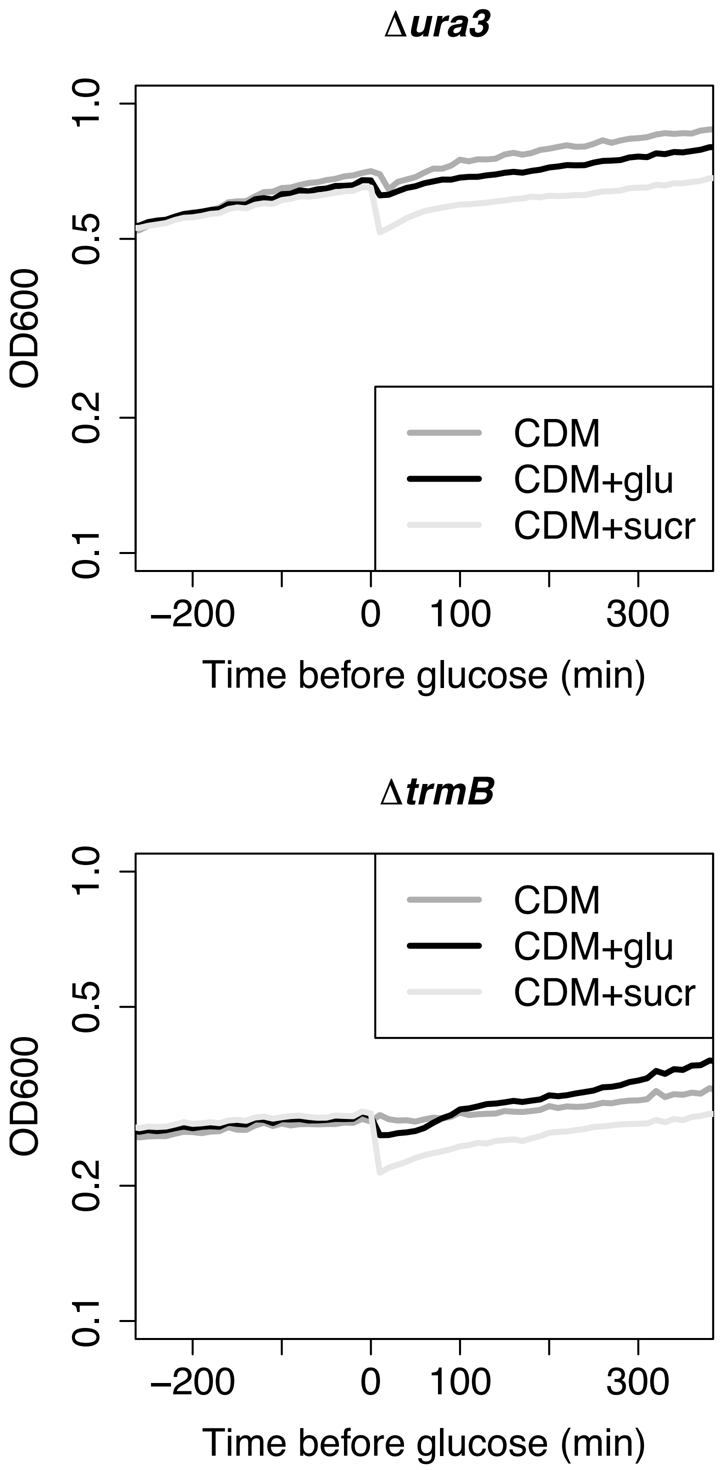

Supplementary Figure 3. Optical density over the course of glucose additon. Mean optical density of 3 biological replicate cultures of the Δ*ura3* parent strain (A) and Δ*trmB* mutant strain (B) during the glucose addition (black lines), control (dark grey lines), and sucrose control (light grey lines) time course. Cells were grown in Complete Defined Medium (CDM).
