# Supplementary material for: Dynamic Metabolite Profiling in an Archaeon Connects Transcriptional Regulation to Metabolic Consequences
Source: PLoS One. 2015 Aug 18;10(8):e0135693. doi: 10.1371/journal.pone.0135693 (PMC4540570; doi:10.1371/journal.pone.0135693)
Supplement: S4 Fig — The expression of the gene encoding adenine phosphoribosyltransferase is similar in both the ΔtrmB mutant and its Δura3 isogenic parent strain in the absence or presence of glucose. Bar plots show log10 gene expression normalized to the Δura3 parent strain in the absence of glucose (data from [8]) in the Δura3 parent strain (dark bars) and ΔtrmB mutant strain (light bars) in the absence and presence of glucose. Error bars represent standard error from the mean of at least 5 microarrays. (PDF) [file pone.0135693.s004.pdf]

Supplementary Figure 4

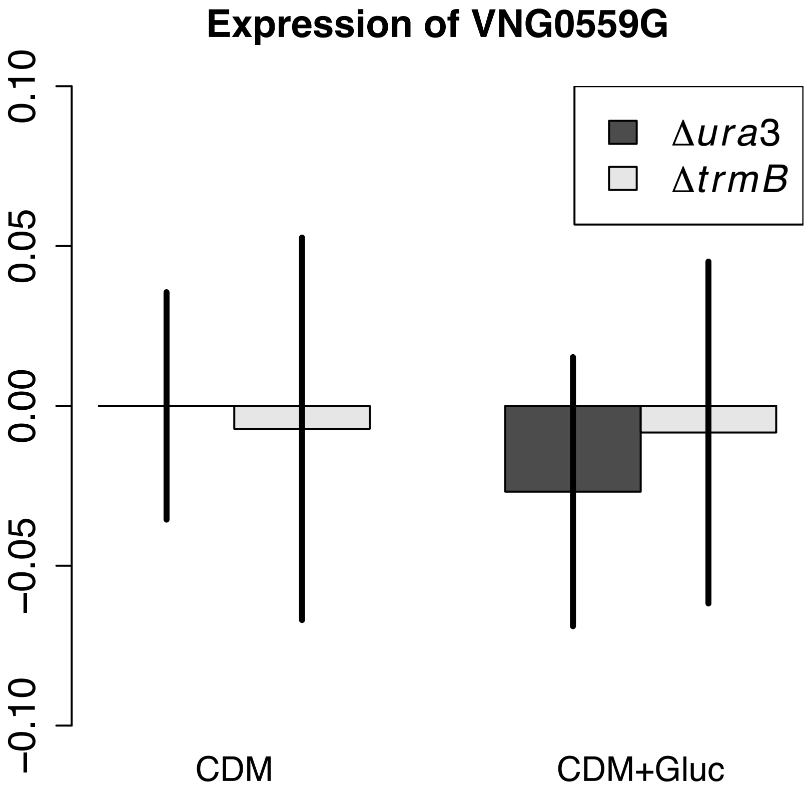

Supplementary Figure 4. Expression of the gene encoding adenine phosphoribosyltransferase in the Δ*ura3* and Δ*trmB* strains with and without glucose. The expression of the gene encoding adenine phosphoribosyltransferase is similar in both the Δ*trmB* mutant and its Δ*ura3* isogenic parent strain in the absence or presence of glucose. Bar plots show log10 gene expression normalized to the Δ*ura3* parent strain in the absence of glucose (data from (8) in the Δ*ura3* parent strain (dark bars) and Δ*trmB* mutant strain (light bars) in the absence and presence of glucose. Error bars represent standard error from the mean of at least 5 microarrays.
